# The Janus-faced role of TBK1 in health and disease: balancing immune defense and pathological progression

**DOI:** 10.3389/fimmu.2026.1995184

**Published:** 2026-10-22

**Authors:** Jingyi Fu, Zhaolong Li

**Affiliations:** 1 Institute of Virology and AIDS Research, The First Hospital of Jilin University, Changchun, Jilin, China; 2 Department of Infectious Diseases, Infectious Diseases and Pathogen Biology Center, Key Laboratory of Organ Regeneration and Transplantation of The Ministry of Education, The First Hospital of Jilin University, Changchun, Jilin, China

**Keywords:** antiviral immunity, autophagy, cancer, inflammation, innate immunity, pathogen infection, TBK1

## Abstract

TANK-binding kinase 1 (TBK1) is a central innate immune kinase that links pathogen recognition to type I interferon production. However, accumulating evidence indicates that its functions extend beyond canonical antiviral signaling to autophagy, mitochondrial quality control, cellular metabolism, cell survival, and cell death. Here, we review the multifaceted roles of TBK1 under physiological and pathological conditions, with emphasis on infectious diseases, cancer, and other non-infectious diseases. During infection, TBK1 integrates signals from multiple pattern recognition receptors to activate IRF3- and NF-κB-dependent host defenses, while diverse pathogens evade immunity by targeting TBK1 or its associated signaling complexes. In cancer, TBK1 exhibits context-dependent effects by supporting tumor cell survival and stress adaptation while also promoting antitumor immunity downstream of STING. Dysregulated TBK1 signaling is further implicated in neurodegenerative, autoinflammatory, and chronic inflammatory diseases, in which either TBK1 activation or loss of function can contribute to pathology. Collectively, these findings indicate that TBK1 should not be simply classified as protective or pathogenic. Its biological outputs are determined by cell type, upstream stimuli, molecular interaction networks, and the spatiotemporal state of signaling. This functional plasticity makes TBK1 a promising but complex therapeutic target. Rather than generalized inhibition or activation, future TBK1-targeted strategies should consider disease mechanisms and cell-specific contexts to achieve precise therapeutic modulation.

## 1 Introduction

The innate immune system serves as the first line of defense against invading pathogens and plays a fundamental role in maintaining internal homeostasis. Host cells sense pathogen-associated molecular patterns (PAMPs) and damage-associated molecular patterns (DAMPs) through pattern recognition receptors (PRRs), thereby initiating complex signaling cascades that induce the production of type I interferons (IFNs) and inflammatory cytokines. Among these, canonical innate immune sensing pathways, including RIG-I-like receptors (RLRs), Toll-like receptors (TLRs), and the cyclic GMP-AMP synthase–stimulator of interferon genes (cGAS–STING) pathway, all depend on the precise regulation of downstream signaling molecules. As a key signaling hub that links multiple pattern recognition receptors to downstream transcription factors, TANK-binding kinase 1 (TBK1) plays an important role in regulating antiviral immunity, inflammatory responses, and immune homeostasis. In recent years, with increasing insights into the functions of TBK1, its biological roles have expanded beyond its canonical function in host defense against infection to encompass the regulation of cellular homeostasis, tumorigenesis and tumor progression, and a broad range of inflammation-associated diseases (1, 2).

TANK-binding kinase 1 (TBK1) is a serine/threonine protein kinase belonging to the IκB kinase (IKK)-related family and serves as a central regulator of multiple innate immune signaling pathways. The canonical function of TBK1 is primarily characterized by its role in mediating pattern recognition receptor (PRR)-induced type I interferon production. During RNA virus infection, RIG-I-like receptors (RLRs) recognize viral RNA and recruit and activate TBK1 through mitochondrial antiviral-signaling protein (MAVS), thereby promoting the phosphorylation and dimerization of interferon regulatory factor 3 (IRF3), which subsequently induces the expression of type I interferons and interferon-stimulated genes (ISGs). In addition, during cytosolic DNA sensing, the cyclic GMP-AMP synthase–stimulator of interferon genes (cGAS–STING) pathway also relies on TBK1-mediated activation of IRF3 to initiate antiviral immune responses. However, the functions of TBK1 are not limited to promoting protective immunity. Increasing evidence has demonstrated that TBK1 also participates in the regulation of NF-κB-mediated inflammatory responses, autophagy, cell survival, and other biological processes, with its functional outcomes being highly dependent on cell type, stimulatory conditions, and disease context. Thus, TBK1 is not only an essential regulator of host immune defense but may also exert complex and context-dependent dual effects in chronic inflammation, pathogen immune evasion, and tumorigenesis and tumor progression (3, 4).

The activity and signaling output of TBK1 are regulated by multiple layers of molecular control. TBK1 contains an N-terminal kinase domain, a ubiquitin-like domain (ULD), a scaffold and dimerization domain (SDD), and a C-terminal adaptor-binding region. Phosphorylation of TBK1 at Ser172 is a critical step in its activation, while ubiquitination and oligomerization further regulate TBK1 activation and signaling. In addition, adaptor proteins including TANK, NAP1, and SINTBAD recruit TBK1 into distinct signaling complexes and facilitate its activation at specific subcellular sites, thereby contributing to the regulation of downstream substrate selection and biological outputs (5–7).

Several recent reviews have discussed TBK1-mediated autophagy, cancer immunobiology, its paradoxical roles in tumor development, and cell type-specific immune regulation (8–11). However, these studies have largely focused on individual biological processes or disease contexts.

In this review, we examine the functions of TBK1 across infectious, malignant, neurodegenerative, and inflammatory diseases using a mechanistic framework. Rather than simply categorizing TBK1 as protective or pathogenic, we focus on how cellular compartment, signaling complex or adaptor, substrate selection, and disease stage shape the biological outcomes of TBK1 signaling. By comparing these determinants across disease contexts, we aim to clarify the mechanisms underlying the divergent functions of TBK1 and to provide a framework for understanding its context-dependent therapeutic potential.

## 2 Physiological functions of TBK1

Under normal physiological conditions, TBK1 serves primarily as a critical hub in innate immune signal transduction and participates in the basal surveillance of aberrant nucleic acids and danger signals. Signals originating from different pattern recognition receptors (PRRs) can ultimately converge on TBK1, whose kinase activity promotes the phosphorylation and dimerization of IRF3 and IRF7, thereby inducing the expression of type I interferons (IFN-I) and related interferon-stimulated genes (ISGs) (3, 4). During this process, TBK1 activation is tightly regulated at multiple levels, including ubiquitination, phosphorylation, and the assembly of protein signaling complexes, thereby restricting the magnitude and duration of signaling and preventing basal immune surveillance from developing into persistent inflammation. Thus, the physiological role of TBK1 is not simply to sustain continuous IFN production, but rather to maintain an appropriate threshold of immune responsiveness through a dynamic process involving danger signal sensing—TBK1 activation—IRF3/7 phosphorylation—IFN-I production—and signal termination.

The clearance of damaged mitochondria is an essential process for maintaining cellular homeostasis, and TBK1 plays a critical role in PINK1/Parkin-dependent mitophagy. Following mitochondrial damage, PINK1 accumulates on the outer mitochondrial membrane and activates Parkin, which subsequently promotes the ubiquitination of mitochondrial outer membrane proteins, generating ubiquitin signals that can be recognized by autophagy receptors (12). These ubiquitin signals facilitate the recruitment of autophagy receptors such as OPTN and NDP52 to damaged mitochondria and induce TBK1 activation in the vicinity of the damaged mitochondria (13). Activated TBK1 subsequently phosphorylates autophagy receptors such as OPTN, enhancing their interactions with ubiquitin chains and LC3/GABARAP family proteins, thereby promoting the recruitment of autophagosomal membranes to damaged mitochondria and facilitating mitophagy (13, 14). Thus, TBK1 does not simply function as a downstream kinase of the PINK1/Parkin pathway; rather, through TBK1-dependent phosphorylation of autophagy receptors, it converts mitochondrial ubiquitination signals into autophagosome recruitment signals, thereby enabling the selective clearance of damaged mitochondria.

In addition to mitophagy, TBK1 is also involved in the formation and maturation of autophagosomes. Studies have shown that TBK1 can phosphorylate the autophagy receptor p62/SQSTM1, thereby modulating its interactions with ubiquitinated substrates and autophagy-associated structures and promoting autophagosome maturation. In this process, TBK1 activation functionally couples immune signaling with the autophagy pathway, enabling cells to link the recognition of pathogens or other aberrant intracellular components to their subsequent autophagic clearance (15). Thus, TBK1-mediated regulation of autophagy is not independent of its immune functions but may instead represent a cellular response mechanism that integrates danger sensing, selective autophagy, and cargo clearance.

Taken together, TBK1 links at least two interconnected cellular processes under physiological conditions. On the one hand, TBK1 maintains innate immune surveillance through IRF3/7-mediated production of type I interferons (IFN-I); on the other hand, it contributes to selective autophagy and mitochondrial quality control through the phosphorylation of autophagy receptors such as OPTN and NDP52 (3, 13–15). Thus, TBK1 can be viewed as a signaling hub that integrates innate immune signaling, organelle quality control, and cellular homeostasis. Notably, this functional versatility also means that the biological effects of TBK1 are highly dependent on the cell type in which signaling occurs, the nature of upstream stimuli, and the duration of signal activation. The same TBK1 signaling module may promote cellular protection and homeostasis under physiological conditions, but can be hijacked by pathogens or tumor cells in the settings of persistent infection, chronic inflammation, or the tumor microenvironment, thereby producing distinct biological consequences. This transition from “physiological homeostasis” to “pathological reprogramming” provides the basis for the subsequent discussion of the roles of TBK1 in infection and cancer.

## 3 TBK1 in pathogen infection

Pathogen infection represents one of the most well-established pathological contexts in which the functions of TBK1 have been investigated. Although viruses, bacteria, and fungi differ in their modes of infection and intracellular life cycles, they can all regulate TBK1 through distinct pattern recognition receptors (PRRs) and downstream signaling platforms. During viral infection, viral RNA or DNA can activate TBK1 through sensing systems such as the RIG-I/MDA5–MAVS and cGAS–STING pathways. TBK1 subsequently phosphorylates IRF3, promoting its dimerization and nuclear translocation and thereby inducing the expression of type I interferons (IFN-I) and interferon-stimulated genes (ISGs), which establishes an intracellular antiviral state (3, 16). Thus, the canonical role of TBK1 in host defense against pathogens is to convert pathogen-derived nucleic acid sensing signals into an IFN-I-mediated transcriptional program. However, the functions of TBK1 during infection extend beyond IFN-I production. Its kinase activity can also be coupled to autophagy, organelle quality control, and other mechanisms of cell-autonomous immunity, positioning TBK1 as an important signaling node that links pathogen sensing to pathogen clearance (8). The major TBK1 signaling pathways and their context-dependent downstream effects are summarized in **Figure 1**.

**Figure 1:**
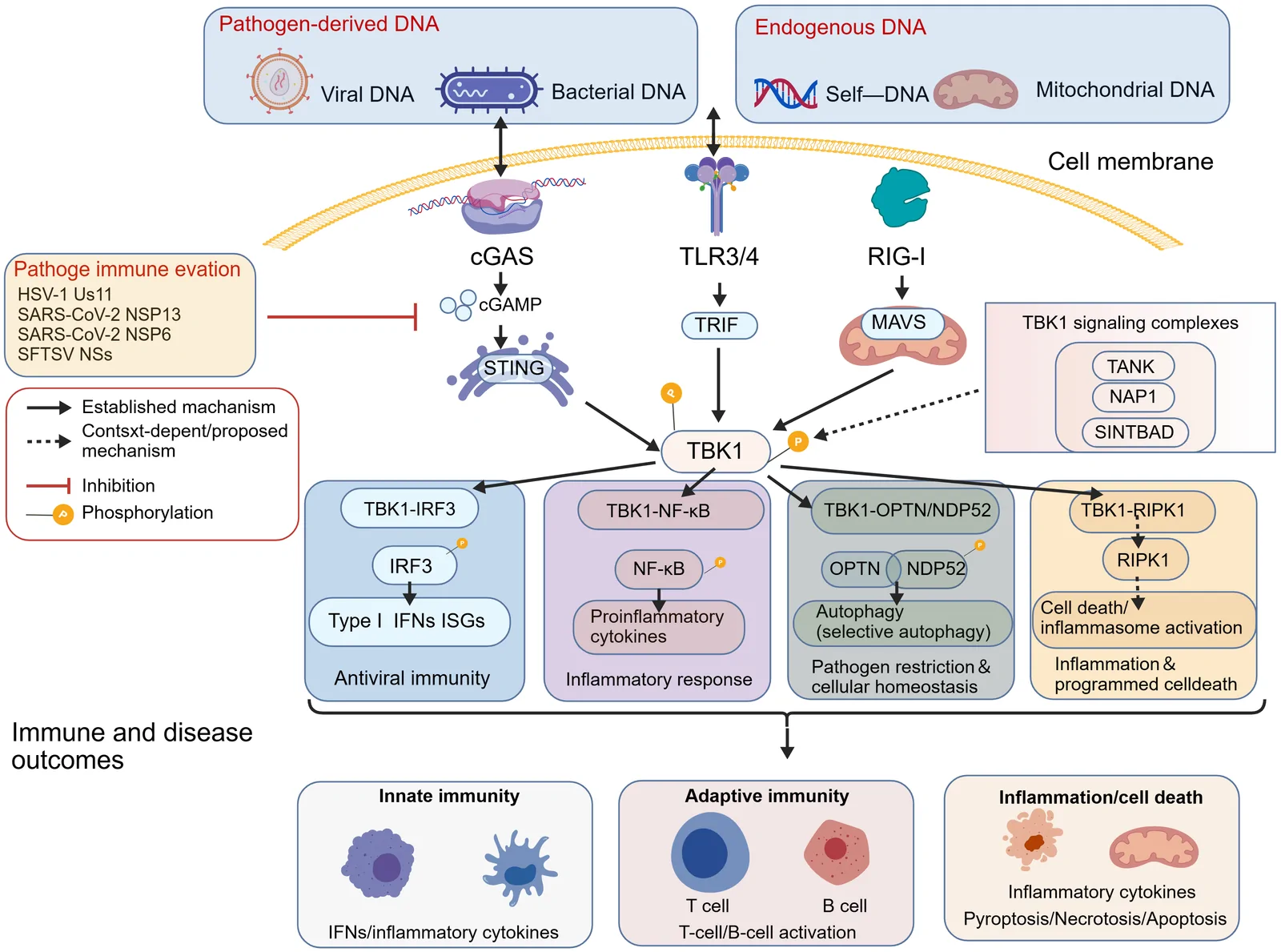
TBK1 signaling in innate immunity and inflammation. Distinct upstream sensing pathways activate TBK1, which regulates IRF3/NF-κB signaling, autophagy, and RIPK1-dependent cell death, thereby contributing to antiviral immunity, inflammation, and immune homeostasis. Solid arrows indicate established mechanisms, whereas dashed arrows indicate context-dependent relationships (Created by BioGDP).

In parallel with the host use of TBK1 to restrict pathogen replication, numerous viruses have evolved immune evasion strategies targeting the TBK1 signaling axis. These mechanisms can act at multiple levels, including TBK1 activation, TBK1–IRF3 signal transduction, and downstream IRF3-dependent transcription. For example, the nonstructural protein NSs of severe fever with thrombocytopenia syndrome virus (SFTSV) sequesters TBK1, IKKϵ, and IRF3 within virus-induced cytoplasmic inclusion bodies, thereby preventing activated IRF3 from efficiently entering the nucleus and suppressing IFN-β production (17). HSV-1 can also target TBK1 through multiple viral proteins. Its γ1(34.5) protein inhibits TBK1 activity and reduces IRF3 phosphorylation, thereby facilitating viral replication (18). In addition, its Us11 protein disrupts the Hsp90–TBK1 complex by binding to Hsp90 and subsequently promotes proteasome-dependent degradation of TBK1, ultimately suppressing IRF3 phosphorylation as well as IFN-β and ISG expression (19). Coronaviruses can likewise block host antiviral responses at the level of TBK1. The M protein of MERS-CoV interacts with TRAF3 and disrupts the formation of the TRAF3–TBK1 complex, thereby inhibiting TBK1-dependent IRF3 activation (20). Collectively, these studies demonstrate that although different viruses employ distinct viral proteins and molecular strategies, their immune evasion mechanisms ultimately converge on several critical steps, including impaired TBK1 activation, disruption of TBK1-containing signaling complexes, reduced TBK1 stability, and inhibition of TBK1–IRF3 signal transduction.

SARS-CoV-2 provides a representative example of the molecular interplay between the host and virus mediated by TBK1. Studies have shown that SARS-CoV-2 NSP6 interacts with TBK1 and inhibits IRF3 phosphorylation, whereas NSP13 can also bind to TBK1 and reduce the activation levels of both TBK1 and IRF3, thereby suppressing type I interferon (IFN-I) production (21). In addition, the SARS-CoV-2 M protein interacts with MDA5, TRAF3, IKKϵ, and TBK1 and induces K48-linked ubiquitination and degradation of TBK1, thereby disrupting the TRAF3–TANK–TBK1–IKKϵ complex and suppressing IFN-I production (22). Notably, the antagonistic effect of NSP13 on the interferon response is not entirely dependent on TBK1. NSP13 can also directly interact with IRF3 and inhibit IRF3-mediated antiviral transcription in a TBK1-independent manner (23). Thus, SARS-CoV-2 antagonism of the TBK1–IRF3 axis occurs at multiple levels, ranging from TBK1 phosphorylation and TBK1 protein stability to IRF3 transcriptional activity, illustrating how viruses can enhance their ability to evade host innate immunity through multi-site interference with antiviral signaling.

Compared with viral infection, the role of TBK1 in bacterial infection more clearly highlights its biological functions beyond its canonical role in IFN-I production. During infection with *Salmonella enterica* serovar Typhimurium, loss of TBK1 facilitates bacterial escape from the pathogen-containing vacuole into the cytosol and increases bacterial replication. This restrictive effect depends on TBK1 kinase activity but is independent of IRF3 and IFN-I, suggesting that TBK1 can maintain the integrity of the bacterial-containing compartment through non-canonical mechanisms (24). Once *Salmonella* enters the cytosol and becomes ubiquitinated, the autophagy receptor NDP52 recognizes ubiquitin chains on the bacterial surface and recruits TBK1 through NAP1 and SINTBAD. At the same time, NDP52 promotes LC3 recruitment, thereby linking TBK1 signaling to selective autophagy and restricting cytosolic bacterial proliferation (25). Further studies have shown that the recruitment of TBK1 to cytosolic invasive *Salmonella* promotes WIPI2-dependent antibacterial autophagy, thereby enhancing the clearance of cytosolic bacteria (26).TBK1 can also serve as a direct target of pathogen-mediated antagonism during fungal infection. A study published in 2024 demonstrated that *Candida albicans* delivers the effector protein Cmi1 into host cells, where Cmi1 directly binds to and inhibits TBK1, thereby reducing IRF3 phosphorylation and IFN-I production. In mouse infection models, deletion of *cmi1* enhanced the IFN-I response, promoted fungal clearance, and improved host survival (27). Thus, from viruses and bacteria to fungi, the role of TBK1 in infection can be conceptualized as a dynamic host–pathogen interplay. The host exploits mechanisms including the TBK1–IRF3/IFN-I axis, TBK1–NDP52–LC3 signaling, and TBK1-mediated maintenance of pathogen-containing compartment integrity to restrict pathogen replication and promote pathogen clearance, whereas pathogens can reprogram this defense network by altering TBK1 localization, protein–protein interactions, phosphorylation status, protein stability, or downstream IRF3 activity (17–27). The major TBK1-targeting mechanisms during pathogen infection and their biological consequences are summarized in **Table 1**.

**Table 1:** TBK1-targeting mechanisms and biological consequences during pathogen infection.

Pathogen	Pathogen factor	TBK1-targeting/TBK1-mediated mechanism	Downstream molecular event	Biological consequence	Ref.
SFTSV	NSs	Sequesters TBK1/IKKϵ/IRF3 into inclusion bodies	↓IRF3 nuclear translocation	↓IFN-β, ↑viral replication	(17)
HSV-1	γ1(34.5)	Inhibits TBK1	↓IRF3 phosphorylation	↑viral replication/neuroinvasion	(18)
HSV-1	Us11	Disrupts Hsp90–TBK1 complex; TBK1 destabilization	↓IRF3/IFN response	↑viral replication	(19)
SARS-CoV-2	NSP13	Interacts with TBK1; inhibits TBK1/IRF3 activation	↓IFN-I	Immune evasion	(21)
SARS-CoV-2	NSP6	Binds TBK1	↓IRF3 phosphorylation	↓IFN-I	(21)
S. Typhimurium	–	NDP52 recruits TBK1 to ubiquitinated bacteria	LC3/autophagy	↓bacterial proliferation	(25)
S. Typhimurium	–	TBK1 recruitment → WIPI2	Antibacterial autophagy	Bacterial clearance	(26)
Salmonella/EPEC/S. pyogenes	–	TBK1 kinase activity maintains pathogen-containing vacuole integrity	Restriction of cytosolic escape	↓bacterial replication	(24)
Candida albicans	Cmi1	Direct TBK1 inhibition	↓IRF3 phosphorylation, ↓IFN-I	Fungal persistence	(27)

## 4 TBK1 in cancer

The role of TBK1 in cancer is closely associated with oncogenic RAS signaling, particularly in KRAS-driven tumors. Systematic RNA interference screening identified TBK1 as an important selective dependency of oncogenic KRAS-driven cancer cells, and TBK1 loss markedly impaired the survival of KRAS-mutant tumor cells, accompanied by reduced NF-κB activity. Further studies demonstrated that TBK1 sustains the survival of RAS-driven tumor cells by promoting NF-κB-associated anti-apoptotic programs, thereby rendering these cells more sensitive to apoptotic stimuli upon TBK1 loss (28). In addition to NF-κB signaling, TBK1 can also directly link oncogenic signaling to the AKT survival pathway. TBK1 can be recruited to the exocyst complex and promote AKT phosphorylation, thereby enhancing AKT-dependent cell survival and malignant transformation, and this process is not entirely dependent on canonical PDK1- or mTORC2- mediated AKT activation (29). In lung cancer, TBK1 further exhibits context-selective support of the activated AKT/mTOR pathway, particularly in tumor settings with aberrant activation of the RAS pathway, where TBK1 activity sustains AKT/mTORC1 signaling and promotes tumor cell survival (30). Thus, in RAS-driven cancers, TBK1 can connect oncogenic RAS signaling with NF-κB- and AKT/mTOR-mediated cell survival programs, making TBK1 a potential vulnerability of tumor cells with specific genetic backgrounds.

Beyond the canonical NF-κB and AKT survival pathways, TBK1 may also help tumor cells adapt to metabolic stress through selective autophagy and mitochondrial quality control. TBK1 can phosphorylate the selective autophagy receptor OPTN, thereby enhancing the binding of OPTN to ubiquitin chains and promoting the selective autophagic clearance of damaged mitochondria, thus contributing to mitochondrial quality control (14). This mechanism is relevant to understanding the link between TBK1 and tumor metabolism, as RAS-driven tumor cells are intrinsically highly dependent on autophagy to maintain mitochondrial function, oxidative metabolism, and tumorigenesis (31). Therefore, these findings provide a plausible rationale for a potential link between TBK1-mediated selective autophagy and metabolic adaptation in RAS-driven tumors. However, whether TBK1-dependent mitophagy directly contributes to metabolic adaptation in RAS-driven tumor cells remains to be established. A more appropriate interpretation is that TBK1 may contribute to stress adaptation in certain tumor cells by linking selective autophagy and mitochondrial quality control with oncogenic signaling, although this proposed connection requires direct validation in relevant cancer models.

Meanwhile, the role of TBK1 in cancer is not limited to tumor cells themselves, as TBK1 is also a critical effector kinase in cGAS–STING-mediated antitumor innate immunity. When tumor cells undergo genomic instability, DNA damage, or mitochondrial damage, cytosolic DNA can be sensed by cGAS, leading to the production of cGAMP, which subsequently activates STING and recruits TBK1. TBK1 then phosphorylates IRF3 and promotes the production of type I interferons (IFN-I). In addition to IRF3, STING-mediated recruitment of TBK1 can also promote NF-κB activation, thereby inducing the expression of inflammatory cytokines and chemokines and facilitating dendritic cell activation and subsequent T cell-mediated antitumor immunity (32). Structural studies have further demonstrated that STING can directly form a complex with TBK1 and promote TBK1 clustering and activation, thereby facilitating IRF3 phosphorylation and providing a structural basis for the transmission of STING signaling to downstream transcriptional responses (33). Thus, the functions of TBK1 in cancer exhibit pronounced cell-context dependence: within tumor cells, TBK1 can support RAS dependent NF-κB and AKT/mTOR survival signaling, whereas within the tumor immune microenvironment, TBK1 serves as a critical node in STING–IRF3-mediated antitumor innate immunity.

## 5 TBK1 in other diseases

The link between TBK1 dysfunction and neurodegenerative diseases provides important evidence for understanding its roles in non-infectious diseases. Genetic studies have revealed that heterozygous loss-of-function mutations in TBK1 are closely associated with the development of amyotrophic lateral sclerosis (ALS) and frontotemporal dementia (FTD), suggesting that TBK1 haploinsufficiency itself can represent an important pathogenic factor in neurodegenerative diseases. Mechanistically, loss of TBK1 function not only impairs its canonical innate immune signaling but may also disrupt the functional connection between TBK1 and the autophagy receptor OPTN, thereby impairing the autophagic clearance of damaged organelles and protein cargoes (34). Further studies have demonstrated that TBK1 participates in the regulation of autophagy and endolysosomal pathways in neurons, and its loss of function can lead to aberrant phosphorylation of autophagy-related proteins, impaired RAB7A-associated late endosomal function, and lysosomal dysfunction, ultimately resulting in a cell-autonomous neurodegenerative phenotype (35, 36). These findings suggest that, in neurodegenerative diseases such as ALS and FTD, TBK1 deficiency may not cause disease simply by weakening antiviral interferon responses, but rather by disrupting the TBK1–OPTN/SQSTM1–autophagy and endolysosomal pathways, thereby progressively compromising proteostasis and organelle quality control and increasing neuronal susceptibility to chronic damage and degeneration (34, 35).

In addition to impaired autophagy, TBK1 deficiency may promote neurodegenerative pathology by releasing its inhibitory control over RIPK1-dependent cell death and inflammatory responses. Studies have shown that TBK1 is an important endogenous inhibitor of RIPK1. Beyond RIPK1-associated apoptosis and inflammatory cell death, emerging evidence suggests that TBK1 may also influence ferroptosis and cellular iron homeostasis. TBK1 inhibition has been reported to promote ferroptosis through the p62–KEAP1–NRF2 axis, while TBK1 has also been implicated in BACH1-dependent regulation of iron homeostasis in cancer cells. These findings further indicate that TBK1 may regulate cell fate through multiple forms of regulated cell death in a context-dependent manner (37, 38). In the context of TBK1 haploinsufficiency, reduced expression of TAK1, another endogenous inhibitor of RIPK1, during aging may further enhance RIPK1 activity and promote ALS/FTD-associated pathological changes, including neuroinflammation, TDP-43 aggregation, axonal degeneration, and neuronal loss (39). Notably, the role of TBK1 in neurodegenerative diseases may also exhibit marked cell-type specificity. Recent studies have shown that TBK1 deficiency can substantially alter microglial homeostasis and reactivity, driving microglia toward a pro-inflammatory “primed” state characterized by features of aging and neurodegeneration, which is sufficient to induce FTD-like deficits in social recognition (40). Thus, TBK1-associated neurodegenerative diseases may not result from dysregulation of a single pathway, but rather from the convergence of multiple defects, including neuronal autophagy/endolysosomal dysfunction and microglial inflammatory dysregulation. In other words, the importance of TBK1 in the nervous system may lie in its ability to simultaneously maintain protein and organelle homeostasis in neurons while restraining excessive inflammatory responses in glial cells. When both of these protective mechanisms are compromised, neurodegenerative pathology may be further amplified.

TBK1 dysfunction is also closely associated with autoinflammatory and chronic inflammatory diseases, with its roles again exhibiting complex bidirectional effects. On the one hand, TBK1 is an important downstream kinase in multiple DNA- and RNA-sensing pathways, and sustained TBK1 activation can promote IRF3-dependent type I interferon responses and further influence cellular metabolic states. For example, in a model of chronic innate immune activation caused by Trex1 deficiency, persistent activation of the cGAS–STING–TBK1 signaling axis suppresses mTORC1 activity and leads to substantial alterations in mitochondrial respiration, energy expenditure, and lipid metabolism, revealing a link between the TBK1–mTORC1 axis and metabolic dysregulation during chronic inflammation (41). On the other hand, complete loss of TBK1 function in humans can also cause severe systemic autoinflammation. This phenotype is not primarily characterized by a complete loss of interferon responses, but rather by enhanced TNF-induced RIPK1-dependent cell death; notably, anti-TNF therapy can substantially ameliorate the inflammatory state in affected patients (42). Therefore, in non-infectious inflammatory diseases, TBK1 may contribute to chronic inflammation through IFN-related signaling and metabolic reprogramming when persistently activated, whereas TBK1 loss of function may trigger autoinflammation by relieving its inhibitory control over TNF–RIPK1-mediated cell death. This phenomenon, in which both excessive activation and loss of function can be pathogenic, further demonstrates that the disease-associated effects of TBK1 depend on the strength and duration of its signaling as well as the cellular context in which it operates. Maintaining an appropriate dynamic balance of TBK1 signaling may therefore be more important than simply enhancing or inhibiting TBK1 activity. The major disease contexts associated with TBK1 dysregulation and their underlying molecular mechanisms are summarized in **Table 2**.

**Table 2:** TBK1 dysregulation and molecular mechanisms in other diseases.

Disease category	TBK1 status	Key molecular mechanism	Major outcome	Ref.
ALS/FTD	Loss-of-function	TBK1 haploinsufficiency; impaired OPTN interaction	Neurodegeneration	(34)
ALS/FTD	TBK1 deficiency	Autophagy/endolysosomal dysfunction; RAB7A dysregulation	Neurite and lysosomal defects	(36)
ALS/FTD	TBK1 haploinsufficiency with reduced TAK1	Increased RIPK1 activity	Neuroinflammation, TDP-43 pathology and neuronal loss	(39)
FTD-like phenotype	Microglial TBK1 loss	Aged-like, pro-inflammatory microglial state	Social recognition deficits	(40)
Chronic inflammatory disease	Sustained TBK1 activation	TBK1–mTORC1 axis	Metabolic dysregulation	(41)
Autoinflammation	Complete TBK1 loss	TNF–RIPK1-dependent cell death	Systemic inflammation	(42)

## 6 Therapeutic targeting of TBK1

The therapeutic appeal of TBK1 as a target primarily stems from its involvement in multiple disease-related processes, including oncogenic signaling, inflammatory responses, autophagy, and cell death. However, the broad functional spectrum of TBK1 also means that its therapeutic targeting cannot be simply interpreted as a generalized strategy of kinase inhibition. In certain KRAS-driven tumors, TBK1 serves as an important signaling node that sustains tumor cell survival, and TBK1 inhibition can attenuate NF-κB- and AKT-associated pro-survival programs, thereby providing a potential therapeutic strategy for targeting RAS-driven tumors (28, 29). Based on this rationale, several small-molecule TBK1 inhibitors have been developed for research and therapeutic exploration, including BX795, MRT67307, Compound II, GSK8612, and amlexanox. However, early TBK1 inhibitors generally suffered from limited selectivity. For example, in addition to inhibiting TBK1 and IKKϵ, BX795 also targets other kinases, meaning that its observed antitumor effects cannot be attributed entirely to TBK1 inhibition (43). This issue highlights that a key challenge for future TBK1-targeted therapeutics is not merely to achieve greater inhibitory potency, but also to improve selectivity for TBK1 and determine whether the observed drug effects are attributable to TBK1 itself or to other off-target kinases.

In cancer therapy, TBK1 inhibition may be more suitable as a component of combination therapies rather than as a monotherapy. In RAS-driven tumors, TBK1 supports tumor-cell survival through NF-κB- and AKT-associated signaling, providing a rationale for combining TBK1 inhibition with other targeted therapies (28, 29). In addition, TBK1 participates in the regulation of RIPK1 downstream of the TNF receptor, and TBK1 inhibition can increase the sensitivity of tumor cells to TNFα/IFNγ-induced cell death. Sun et al. further showed that TBK1 targeting enhances the response to PD-1 blockade by lowering the cytotoxicity threshold to TNFα/IFNγ, suggesting that TBK1 inhibition can affect both tumor-cell-intrinsic death signaling and tumor–immune interactions. However, the net effects of systemic TBK1 inhibition may depend on the relative contribution of TBK1 in tumor cells and immune cells within the tumor microenvironment (44). Importantly, studies targeting the TBK1/IKKϵ axis have shown that disruption of RIPK1 phosphorylation can enhance tumor-cell susceptibility to killing by CD8+ T cells and NK cells. Because these studies involve combined TBK1/IKKϵ perturbation, their effects should not be attributed exclusively to TBK1 (45).

Beyond cancer, TBK1-targeted strategies also hold potential in chronic inflammatory and autoinflammatory diseases, although their therapeutic rationale differs from that in cancer. In disease states characterized by persistent aberrant TBK1 activation, pharmacological inhibition of TBK1 can reduce IRF3-dependent type I interferon signaling and may alleviate inflammatory responses driven by aberrant innate immune activation. For example, the TBK1 inhibitor Compound II suppresses TBK1-mediated IFN responses and ameliorates the autoimmune disease phenotype in Trex1^−^/^−^ mice, improving animal survival while reducing the expression of IFN-related genes in cells from patients with TREX1 mutations (46). However, TBK1 is not a target that can be directly inhibited across all inflammatory diseases, because it also plays an important role in restraining TNF–RIPK1-mediated cell death and inflammation. Complete loss of TBK1 function can lead to aberrant TNF-induced cell death and severe autoinflammation; therefore, further inhibition of TBK1 in diseases associated with TBK1 insufficiency may produce the opposite therapeutic outcome (42). Thus, in inflammatory diseases, the key issue for TBK1-targeted therapy is not simply whether “TBK1 inhibition is effective,” but rather to first determine whether the disease is driven by persistent aberrant TBK1 activation or TBK1 functional deficiency.

It is noteworthy that the greatest challenge for TBK1-targeted therapy arises from its dual, and even opposing, functions across different cell types and disease contexts. On the one hand, TBK1 inhibition can attenuate KRAS-dependent tumor survival signaling and enhance the sensitivity of tumor cells to immune cell-mediated killing (28, 44); on the other hand, TBK1 is also a critical signaling node for cGAS–STING-mediated IRF3 activation and type I interferon production, and therefore broad inhibition of TBK1 could theoretically impair antitumor innate immune responses in certain contexts (32). Similarly, in neurodegenerative diseases, TBK1 insufficiency is closely associated with dysregulation of autophagy, endolysosomal pathways, and RIPK1 regulation, indicating that TBK1 inhibition is unlikely to be appropriate for diseases in which TBK1 loss of function represents a major pathological basis (34, 39). Therefore, future TBK1-targeted therapeutic strategies need to move beyond conventional “kinase inhibition” toward mechanism-guided precision intervention. For TBK1-dependent tumors, selective TBK1 inhibition could be considered in combination with MEK inhibitors or immune checkpoint inhibitors; for autoinflammatory diseases driven by persistent TBK1 activation, TBK1 inhibition may help reduce aberrant interferon signaling; whereas for diseases associated with TBK1 loss of function, therapeutic strategies may instead need to restore TBK1-related functions or target downstream dysregulated pathways, such as RIPK1. Taken together, TBK1 is not a therapeutic target suitable for a “one-size-fits-all” strategy, and its clinical application should ultimately be guided by the TBK1 activity state, disease context, and cellular compartment. The therapeutic potential of TBK1 lies not in universal inhibition or activation, but in matching the direction of TBK1 modulation to its disease- and cell-specific functional state.

## 7 Discussion

TBK1 was initially regarded primarily as a key innate immune kinase that links pattern recognition receptors to type I interferon production. However, accumulating evidence in recent years has demonstrated that its biological functions extend far beyond canonical antiviral signaling. TBK1 not only participates in pathogen-induced activation of IRF3 and NF-κB, but also contributes to the clearance of damaged mitochondria through phosphorylation of selective autophagy receptors such as OPTN, and regulates cell death and inflammatory responses by restraining aberrant RIPK1 activation (14, 39). Thus, TBK1 lies at the intersection of multiple biological processes, including innate immunity, autophagy, cell death, and organelle homeostasis. Across different disease contexts, this functional versatility can produce markedly distinct, and even opposing, biological consequences: pathogens can evade host immunity by inhibiting or hijacking TBK1 signaling; certain KRAS-driven tumors depend on TBK1 to sustain NF-κB- and AKT-associated survival programs (28, 29); meanwhile, TBK1 also participates in STING-dependent antitumor innate immune responses (32).However, current discussions of the “pathogenic” or “protective” roles of TBK1 remain somewhat oversimplified. Many studies have primarily relied on individual cell models, specific genetic backgrounds, or systemic gene knockout animal models; therefore, the TBK1 phenotypes observed in these settings may not necessarily be directly extrapolated to other tissues or disease environments. Importantly, the functional output of TBK1 is not determined solely by its kinase activity. Cellular compartment, the signaling complex or adaptor that recruits TBK1, substrate selection, and disease stage may collectively determine the biological outcome of TBK1 signaling. The distinct signaling complexes formed by TBK1 with OPTN, STING, RIPK1, and other substrates or adaptor proteins, together with its subcellular localization and the duration of its activation, may all contribute to determining the ultimate signaling output.

These insights also pose important challenges for the therapeutic development of TBK1-targeted strategies. Existing studies have demonstrated that TBK1 inhibition can impair tumor cell survival in certain TBK1-dependent cancers and, under specific conditions, enhance tumor sensitivity to immunotherapy (29, 44). However, most currently available TBK1 inhibitors remain at the preclinical stage, and their selectivity, pharmacokinetic properties, and the safety of long-term systemic TBK1 inhibition require further validation. More importantly, because TBK1 also participates in STING-mediated antitumor immunity, while TBK1 loss of function is associated with ALS/FTD and RIPK1-driven inflammatory pathology (32, 34, 39), broad and sustained inhibition of TBK1 may produce biological consequences opposite to those intended. Therefore, future TBK1-targeted therapies should not be based simply on a single indicator, such as elevated TBK1 expression or TBK1 activation, but should instead integrate its kinase activity, upstream drivers, downstream substrate spectrum, and cell type-specific functions. Key areas for future development include establishing biomarkers capable of distinguishing functionally distinct TBK1 pools, developing more selective TBK1 modulators, and exploring cell type- or disease stage-specific intervention strategies. Overall, the complexity of TBK1 does not imply a lack of therapeutic value. On the contrary, its central position at the intersection of immune signaling, cellular homeostasis, and disease adaptation makes TBK1 a promising therapeutic node that warrants further investigation. The therapeutic potential of TBK1 lies not in universal inhibition or activation, but in matching the direction of TBK1 modulation to its disease- and cell-specific functional state.

## References

[1] Kawai T Akira S . The role of pattern-recognition receptors in innate immunity: update on Toll-like receptors. Nat Immunol. (2010) 11:373–84. doi: 10.1038/ni.1863 20404851

[2] Takeuchi O Akira S . Pattern recognition receptors and inflammation. Cell. (2010) 140:805–20. doi: 10.1016/j.cell.2010.01.022 20303872

[3] Fitzgerald KA McWhirter SM Faia KL Rowe DC Latz E Golenbock DT . IKKepsilon and TBK1 are essential components of the IRF3 signaling pathway. Nat Immunol. (2003) 4:491–6. doi: 10.1038/ni921 12692549

[4] Sharma S tenOever BR Grandvaux N Zhou GP Lin R Hiscott J . Triggering the interferon antiviral response through an IKK-related pathway. Science. (2003) 300:1148–51. doi: 10.1126/science.1081315 12702806

[5] Ryzhakov G Randow F . SINTBAD, a novel component of innate antiviral immunity, shares a TBK1-binding domain with NAP1 and TANK. EMBO J. (2007) 26:3180–90. doi: 10.1038/sj.emboj.7601743 17568778 PMC1914091

[6] Tu D Zhu Z Zhou AY Yun CH Lee KE Toms AV . Structure and ubiquitination-dependent activation of TANK-binding kinase 1. Cell Rep. (2013) 3:747–58. doi: 10.1016/j.celrep.2013.01.033 23453972 PMC3863638

[7] Ma X Helgason E Phung QT Quan CL Iyer RS Lee MW . Molecular basis of Tank-binding kinase 1 activation by transautophosphorylation. Proc Natl Acad Sci USA. (2012) 109:9378–83. doi: 10.1073/pnas.1121552109 22619329 PMC3386122

[8] Herhaus L . TBK1 (TANK-binding kinase 1)-mediated regulation of autophagy in health and disease. Matrix Biol. (2021) 100-101:84–98. doi: 10.1016/j.matbio.2021.01.004 33454423

[9] Miranda A Shirley CA Jenkins RW . Emerging roles of TBK1 in cancer immunobiology. Trends Cancer. (2024) 10:531–40. doi: 10.1016/j.trecan.2024.02.007 38519366 PMC11168882

[10] Wang B Zhang F Wu X Ji M . TBK1 is paradoxical in tumor development: a focus on the pathway mediating IFN-I expression. Front Immunol. (2024) 15:1433321. doi: 10.3389/fimmu.2024.1433321 39161768 PMC11330819

[11] Almeida MS Bezbradica JS Coban C . TANK-binding kinase 1 (TBK1): unexpected cell type-specific immune regulation beyond antiviral type I interferon signaling. Curr Opin Virol. (2026) 76:101545. doi: 10.1016/j.coviro.2026.101545 42054941

[12] Lazarou M Sliter DA Kane LA Sarraf SA Wang C Burman JL . The ubiquitin kinase PINK1 recruits autophagy receptors to induce mitophagy. Nature. (2015) 524:309–14. doi: 10.1038/nature14893 26266977 PMC5018156

[13] Heo JM Ordureau A Paulo JA Rinehart J Harper JW . The PINK1-PARKIN mitochondrial ubiquitylation pathway drives a program of OPTN/NDP52 recruitment and TBK1 activation to promote mitophagy. Mol Cell. (2015) 60:7–20. doi: 10.1016/j.molcel.2015.08.016 26365381 PMC4592482

[14] Richter B Sliter DA Herhaus L Stolz A Wang C Beli P . Phosphorylation of OPTN by TBK1 enhances its binding to Ub chains and promotes selective autophagy of damaged mitochondria. Proc Natl Acad Sci USA. (2016) 113:4039–44. doi: 10.1073/pnas.1523926113 27035970 PMC4839414

[15] Pilli M Arko-Mensah J Ponpuak M Roberts E Master S Mandell MA . TBK-1 promotes autophagy-mediated antimicrobial defense by controlling autophagosome maturation. Immunity. (2012) 37:223–34. doi: 10.1016/j.immuni.2012.04.015 22921120 PMC3428731

[16] Dixit E Boulant S Zhang Y Lee ASY Odendall C Shum B . Peroxisomes are signaling platforms for antiviral innate immunity. Cell. (2010) 141:668–81. doi: 10.1016/j.cell.2010.04.018 20451243 PMC3670185

[17] Wu X Qi X Qu B Zhang Z Liang M Li C . Evasion of antiviral immunity through sequestering of TBK1/IKKϵ/IRF3 into viral inclusion bodies. J Virol. (2014) 88:3067–76. doi: 10.1128/jvi.03510-13 24335286 PMC3957960

[18] Ma Y Jin H Valyi-Nagy T Cao Y Yan Z He B . Inhibition of TANK binding kinase 1 by herpes simplex virus 1 facilitates productive infection. J Virol. (2012) 86:2188–96. doi: 10.1128/jvi.05376-11 22171259 PMC3302378

[19] Liu X Main D Ma Y He B . Herpes simplex virus 1 inhibits TANK-binding kinase 1 through formation of the Us11-Hsp90 complex. J Virol. (2018) 92. doi: 10.1128/jvi.00402-18 29743370 PMC6026733

[20] Lui PY Wong LY Fung CL Siu KL Yeung ML Yuen KS . Middle East respiratory syndrome coronavirus M protein suppresses type I interferon expression through the inhibition of TBK1-dependent phosphorylation of IRF3. Emerg Microbes Infect. (2016) 5:e39. doi: 10.1038/emi.2016.33 27094905 PMC4855074

[21] Xia H Cao Z Xie X Zhang X Chen JY Wang H . Evasion of Type I interferon by SARS-CoV-2. Cell Rep. (2020) 33:108234. doi: 10.1016/j.celrep.2020.108234 32979938 PMC7501843

[22] Sui L Zhao Y Wang W Wu P Wang Z Yu Y . SARS-CoV-2 membrane protein inhibits type I interferon production through ubiquitin-mediated degradation of TBK1. Front Immunol. (2021) 12:662989. doi: 10.3389/fimmu.2021.662989 34084167 PMC8168463

[23] Feng K Zhang HJ Min YQ . SARS-CoV-2 NSP13 interacts with host IRF3, blocking antiviral immune responses. J Med Virol. (2023) 95:e28881. doi: 10.1002/jmv.28881 37314155

[24] Radtke AL Delbridge LM Balachandran S Barber GN O'Riordan MX . TBK1 protects vacuolar integrity during intracellular bacterial infection. PloS Pathog. (2007) 3:e29. doi: 10.1371/journal.ppat.0030029 17335348 PMC1808071

[25] Thurston TL Ryzhakov G Bloor S von Muhlinen N Randow F . The TBK1 adaptor and autophagy receptor NDP52 restricts the proliferation of ubiquitin-coated bacteria. Nat Immunol. (2009) 10:1215–21. doi: 10.1038/ni.1800 19820708

[26] Thurston TLM Boyle KB Allen M Ravenhill BJ Karpiyevich M Bloor S . Recruitment of TBK1 to cytosol-invading Salmonella induces WIPI2-dependent antibacterial autophagy. EMBO J. (2016) 35:1779–92. doi: 10.15252/embj.201694491 27370208 PMC5010046

[27] Luo G Zhang J Wang T Cui H Bai Y Luo J . A human commensal-pathogenic fungus suppresses host immunity via targeting TBK1. Cell Host Microbe. (2024) 32:1536–1551.e6. doi: 10.1016/j.chom.2024.07.003 39084229

[28] Barbie DA Tamayo P Boehm JS Kim SY Moody SE Dunn IF . Systematic RNA interference reveals that oncogenic KRAS-driven cancers require TBK1. Nature. (2009) 462:108–12. doi: 10.1038/nature08460 19847166 PMC2783335

[29] Ou YH Torres M Ram R Formstecher E Roland C Cheng T . TBK1 directly engages Akt/PKB survival signaling to support oncogenic transformation. Mol Cell. (2011) 41:458–70. doi: 10.1016/j.molcel.2011.01.019 21329883 PMC3073833

[30] Cooper JM Ou YH McMillan EA . TBK1 provides context-selective support of the activated AKT/mTOR pathway in lung cancer. Cancer Res. (2017) 77:5077–94. doi: 10.1158/0008-5472.can-17-0829 28716898 PMC5833933

[31] Guo JY Chen HY Mathew R Fan J Strohecker AM Karsli-Uzunbas G . Activated Ras requires autophagy to maintain oxidative metabolism and tumorigenesis. Genes Dev. (2011) 25:460–70. doi: 10.1101/gad.2016311 21317241 PMC3049287

[32] Yum S Li M Fang Y Chen ZJ . TBK1 recruitment to STING activates both IRF3 and NF-κB that mediate immune defense against tumors and viral infections. Proc Natl Acad Sci USA. (2021) 118. doi: 10.1073/pnas.2100225118 33785602 PMC8040795

[33] Zhang C Shang G Gui X Zhang X Bai XC Chen ZJ . Structural basis of STING binding with and phosphorylation by TBK1. Nature. (2019) 567:394–8. doi: 10.1038/s41586-019-1000-2 30842653 PMC6862768

[34] Freischmidt A Wieland T Richter B Ruf W Schaeffer V Müller K . Haploinsufficiency of TBK1 causes familial ALS and fronto-temporal dementia. Nat Neurosci. (2015) 18:631–6. doi: 10.1038/nn.4000 25803835

[35] Mordes DA Smeyers J . TBK1 orchestrates autophagy and endo-lysosomal pathways in human neurons. Autophagy. (2026) 22:632–4. doi: 10.1080/15548627.2025.2609924 41485128 PMC12931900

[36] Smeyers J Oses-Prieto JA Yadanar L Wang M Iadarola M Lu S . Phospho-proteome profiling in human neurons reveals targets of TBK1 in ALS/FTD-associated autophagy networks. Cell Rep. (2025) 44:116494. doi: 10.1016/j.celrep.2025.116494 41171761 PMC12967247

[37] Yang C Lu T Liu M Yuan X Li D Zhang J . Tiliroside targets TBK1 to induce ferroptosis and sensitize hepatocellular carcinoma to sorafenib. Phytomedicine. (2023) 111:154668. doi: 10.1016/j.phymed.2023.154668 36657316

[38] Liu L Matsumoto M Matsui-Watanabe M Ochiai K Callens B Nguyen LC . BACH1 expression is promoted by Tank binding kinase 1 (TBK1) in pancreatic cancer cells to increase iron and reduce the expression of E-cadherin. Antioxidants (Basel). (2022) 11:1460. doi: 10.3390/antiox11081460 36009179 PMC9405201

[39] Xu D Jin T Zhu H Chen H Ofengeim D Zou C . TBK1 suppresses RIPK1-driven apoptosis and inflammation during development and in aging. Cell. (2018) 174:1477–1491.e19. doi: 10.1016/j.cell.2018.07.041 30146158 PMC6128749

[40] Lenoel I Ribon M Lorenc F Diebold A Philibert CE Robaldo D . ALS/FTD-linked TBK1 deficiency in microglia induces an aged-like microglial signature and drives social recognition deficits in mice. Nat Commun. (2025) 16:7951. doi: 10.1038/s41467-025-63211-w 40858618 PMC12381211

[41] Hasan M Gonugunta VK Dobbs N . Chronic innate immune activation of TBK1 suppresses mTORC1 activity and dysregulates cellular metabolism. Proc Natl Acad Sci USA. (2017) 114:746–51. doi: 10.1073/pnas.1611113114 28069950 PMC5278463

[42] Taft J Markson M Legarda D Patel R Chan M Malle L . Human TBK1 deficiency leads to autoinflammation driven by TNF-induced cell death. Cell. (2021) 184:4447–4463.e20. doi: 10.1016/j.cell.2021.07.026 34363755 PMC8380741

[43] Clark K Plater L Peggie M Cohen P . Use of the pharmacological inhibitor BX795 to study the regulation and physiological roles of TBK1 and IkappaB kinase epsilon: a distinct upstream kinase mediates Ser-172 phosphorylation and activation. J Biol Chem. (2009) 284:14136–46. doi: 10.1074/jbc.M109.000414 19307177 PMC2682862

[44] Sun Y Revach OY Anderson S Kessler EA Wolfe CH Jenney A . Targeting TBK1 to overcome resistance to cancer immunotherapy. Nature. (2023) 615:158–67. doi: 10.1038/s41586-023-05704-6 36634707 PMC10171827

[45] Piskopou A Vredevoogd DW Kong X Peeper DS Altelaar M Stecker KE . Inhibition of TBK1/IKKϵ mediated RIPK1 phosphorylation sensitizes tumors to immune cell killing. Cell Death Discov. (2025) 11:551. doi: 10.1101/2025.05.20.655064 41315176 PMC12663160

[46] Hasan M Dobbs N Khan S White MA Wakeland EK Li QZ . Cutting edge: Inhibiting TBK1 by Compound II ameliorates autoimmune disease in mice. J Immunol. (2015) 195:4573–7. doi: 10.4049/jimmunol.1500162 26432890 PMC4635567

